# Condition-specific promoter activities in *Saccharomyces cerevisiae*

**DOI:** 10.1186/s12934-018-0899-6

**Published:** 2018-04-10

**Authors:** Liang Xiong, Yu Zeng, Rui-Qi Tang, Hal S. Alper, Feng-Wu Bai, Xin-Qing Zhao

**Affiliations:** 1 School of Life Science and Biotechnology, Dalian University of Technology, Dalian, 116024 China; 2 State Key Laboratory of Microbial Metabolism (SKLMM), School of Life Sciences and Biotechnology, Shanghai Jiao Tong University, Shanghai, 200240 China; 3 Department of Chemical Engineering, The University of Texas at Austin, Austin, TX 78712 USA

**Keywords:** *Saccharomyces cerevisiae*, Promoter strength, Synthetic promoter, Transcription regulation, Xylose, Environmental stress

## Abstract

**Background:**

*Saccharomyces cerevisiae* is widely studied for production of biofuels and biochemicals. To improve production efficiency under industrially relevant conditions, coordinated expression of multiple genes by manipulating promoter strengths is an efficient approach. It is known that gene expression is highly dependent on the practically used environmental conditions and is subject to dynamic changes. Therefore, investigating promoter activities of *S. cerevisiae* under different culture conditions in different time points, especially under stressful conditions is of great importance.

**Results:**

In this study, the activities of various promoters in *S. cerevisiae* under stressful conditions and in the presence of xylose were characterized using yeast enhanced green fluorescent protein (yEGFP) as a reporter. The stresses include toxic levels of acetic acid and furfural, and high temperature, which are related to fermentation of lignocellulosic hydrolysates. In addition to investigating eight native promoters, the synthetic hybrid promoter *P*_*3xC*-*TEF1*_ was also evaluated. The results revealed that *P*_*TDH3*_ and the synthetic promoter *P*_*3xC*-*TEF1*_ showed the highest strengths under almost all the conditions. Importantly, these two promoters also exhibited high stabilities throughout the cultivation. However, the strengths of *P*_*ADH1*_ and *P*_*PGK1*_, which are generally regarded as ‘constitutive’ promoters, decreased significantly under certain conditions, suggesting that cautions should be taken to use such constitutive promoters to drive gene expression under stressful conditions. Interestingly, *P*_*HSP12*_ and *P*_*HSP26*_ were able to response to both high temperature and acetic acid stress. Moreover, *P*_*HSP12*_ also led to moderate yEGFP expression when xylose was used as the sole carbon source, indicating that this promoter could be used for inducing proper gene expression for xylose utilization.

**Conclusion:**

The results here revealed dynamic changes of promoter activities in *S. cerevisiae* throughout batch fermentation in the presence of inhibitors as well as using xylose. These results provide insights in selection of promoters to construct *S. cerevisiae* strains for efficient bioproduction under practical conditions. Our results also encouraged applications of synthetic promoters with high stability for yeast strain development.

**Electronic supplementary material:**

The online version of this article (10.1186/s12934-018-0899-6) contains supplementary material, which is available to authorized users.

## Background

*Saccharomyces cerevisiae* is commonly used for development of microbial cell factories to produce biofuels and biochemicals. Despite great progress in metabolic engineering and synthetic biology of *S. cerevisiae*, the bioconversion efficiency of the developed strains still requires further optimization, especially under practical application conditions [1]. To improve overall performance of the strains, it is critical to ensure that efficient pathways with balanced gene expression levels are achieved. Besides, the optimal gene expression levels are variable and dependent on different environmental conditions [1–3].

Promoter is one of the most important genetic elements involved in the rational control and optimization of gene expression levels [3]. Activities of different native promoters in *S. cerevisiae* have been characterized [4–6]. In addition, successful examples have been reported to improve production efficiency by fine-tuning gene expression through manipulating multiple promoter strengths [7–12]. With different combinations of promoters to control the expression of pathway genes, combinatorial method has been successful in developing efficient strains [8, 13, 14].

The commonly-used promoters can be divided into two main classes, namely, ‘constitutive’ and ‘inducible’ promoters [3, 15]. ‘Constitutive’ promoters are believed to lead to stable expression throughout varying conditions, whereas ‘inducible’ promoters induce dramatic changes in expression levels in response to environmental stimuli. Strong constitutive promoters that drive high level transcription are often used to achieve high level expression of key enzymes [4, 5]. Among the constitutive promoters, promoters of translational elongation factor EF-1 alpha (*P*_*TEF1*_) and glycolytic genes, such as 3-phosphoglycerate kinase (*P*_*PGK1*_), glyceraldehyde-3-phosphate dehydrogenase (*P*_*TDH3*_), and alcohol dehydrogenase (*P*_*ADH1*_) are commonly utilized [4]. On the other hand, inducible promoters also received increasing attentions for optimization of gene expression. The previous study in our group employed the promoter of *TPS1*, which encodes trehalose-6-phosphate synthase 1, to induce ethanol-responsive expression of *FLO1* [16]. The resultant strain showed optimized flocculation phenotype in response to increasing ethanol concentrations, resulting in significantly improved cell growth and ethanol production titer compared with that of the flocculating strain carrying the constitutive promoter *P*_*PGK1*_. Recently, *HXT1* promoter, which provides both high-glucose induction and low-glucose repression, was also employed to control the expression of key genes for terpenoid synthesis [12, 17, 18]. These studies demonstrated that ‘inducible’ promoters with dynamic activities are powerful for fine control of metabolic outputs during fermentation.

Abundant native promoters in *S. cerevisiae* have been characterized during the past decades [3, 15, 19]. Mutant promoter libraries were also generated to finely modulate expression levels of multiple genes [8, 20, 21]. In addition, artificially synthesized hybrid promoters were constructed by adding tandem upstream activation sequences (UASs) in front of the core promoter elements [22]. Regulatory modules could also be integrated into artificial promoters to allow strict regulation of gene expression [22–24]. Novel synthetic promoters induced by low pH conditions were developed by manipulating transcription binding sites (TFBSs) in the promoter region [24]. Recently, de novo synthetic minimal promoters were also reported [25]. The synthetic promoters provide diverse possibilities to achieve proper gene expression levels under specific conditions.

Besides generating promoter diversities as described above, it is also important to understand the responses of promoter strengths under different conditions. However, related study is still very limited. During the formation of specific products, yeast cells are often subjected to nasty conditions [26]. For example, toxic levels of inhibitors can be released during pretreatment of lignocellulosic biomass [27]. These inhibitors include acetic acid, formic acid, furfural, and so on. In addition, moderately high temperature (35–39 °C) is desired to perform simultaneous saccharification and fermentation (SSF) to relieve inhibition of enzyme activity [26]. So far, the dynamic changes of promoter activities during fermentation under different conditions, especially under stressful environmental conditions, have not been well characterized. To improve production efficiency of microbial cell factories, it is essential to investigate the relationship of promoter activities with different operation conditions.

Due to the gradual depletion of fossil fuels and concerns on environmental protection, production of biofuels and biochemicals derived from renewable lignocellulosic biomass has aroused great research interests. Xylose is the second most abundant sugar in lignocellulosic hydrolysates [28], therefore, rapid and efficient assimilation of xylose is critical for efficient bioconversion of lignocellulosic biomass [29]. Utilization of xylose relies on the cooperation of enzymes encoded by multiple genes, therefore, it is important to characterize the strengths of different promoters during xylose fermentation. In addition to optimizing the expression level of the introduced pathway enzymes, it is also important to manipulate the expression level of the downstream genes [7, 29]. However, studies on the dynamic activities of different promoters are still limited. Transcriptomics and microarray analysis can be employed to hint the activities of different promoters during xylose utilization [5, 30]. Nevertheless, complete profiles of promoter activities can be obtained using reporter systems to avoid limitation of time points in transcriptomics analysis.

Despite extensive studies in development of yeast strains for bioconversion of lignocellulosic biomass, effects of fermentation conditions on promoter activities remain unclear. In this study, the responses of nine promoters, including eight native constitutive promoters and one synthetic hybrid promoter, were investigated using yeast enhanced green fluorescent protein (yEGFP) as a reporter. Special concerns were focused on the responses of the strengths of these promoters under inhibitory conditions and in the presence of xylose. The data presented here provides basis for rational control of gene expression levels under practical fermentation conditions.

## Results

### Construction of the promoter reporter plasmids and quantification of fluorescence

**Fig. 1:**
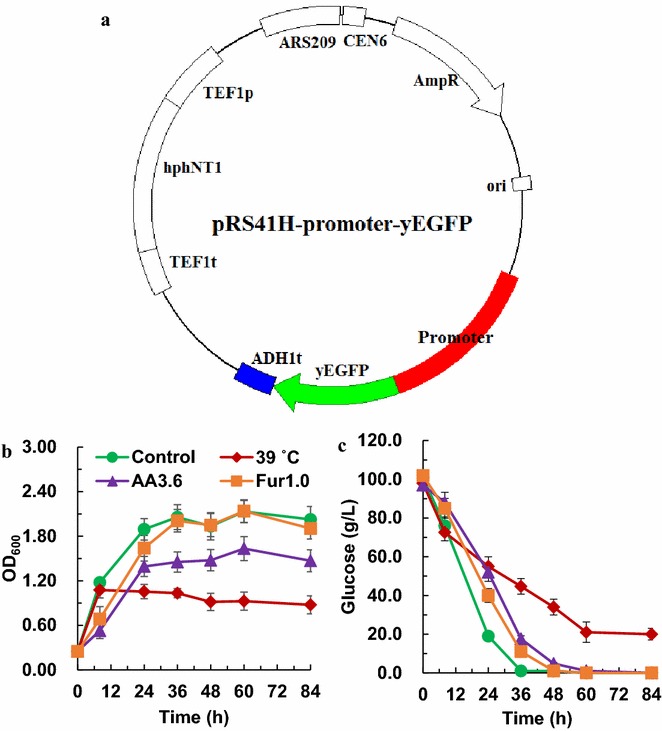
Cell growth of *S. cerevisiae* BY4741 with reporter plasmid under stressful conditions. Map of the reporter plasmid (**a**), cell growth (**b**) and glucose consumption (**c**) of *S. cerevisiae* BY4741 with the reporter plasmid under stressful conditions. *S. cerevisiae* BY4741 derivative strains with the reporter plasmids were cultured in YPD100 medium containing various inhibitors at 30 °C shaking at 200 rpm. Thermal stress treatment was performed at 39 °C. AA3.6 and Fur1.0 represent stressful conditions with, 3.6 g/L acetic acid and 1.0 g/L furfural, respectively. The non-addition group was used as control. Samples at different time points were analyzed and the results were displayed in mean ± standard deviation

The promoter-yEGFP reporter system was established by transforming the reporter centromeric plasmids (Fig. 1a and Table 1) into different *S. cerevisiae* strains, resulting in various reporter strains (Table 2). Subsequently, the promoter activities of *P*_*TDH3*_, *P*_*TEF1*_, *P*_*HSP12*_, *P*_*TPS1*_ and *P*_*3xC*-*TEF1*_ were investigated in the presence or absence of antibiotics (Additional file 1: Figure S1), indicating that the promoter activities revealed by centromeric-plasmid reporter system are relatively stable. The mRNA level of yEGFP under the control of different promoters at log-phase was well correlated with promoter strength determined by quantification of yEGFP fluorescence (Additional file 1: Figure S2), which is consistent with the previous report [22], suggesting that **t**he promoter strengths reflected by fluorescence intensities of yEGFP were convincible. For determination of promoter strength under stressful conditions, *S. cerevisiae* BY4741 was used as a host strain, while the recombinant strain *S. cerevisiae* LX03 with xylose assimilating ability was used as a host for the determination of promoter strength in the presence of xylose. To obtain complete profiles of the promoter strengths under various conditions, four different time points were selected during 84 h for stressful conditions and 72 h for xylose utilization, respectively.

**Table 1:** Plasmids used in this study

Plasmid	Description	Source or references
pAUR-PsXR-PsXDH-ScXK	Plasmid containing tandem xylose assimilating pathway, *AUC*-*1*; *YPRCdelta15::P*_*PGK1*_-*XYL1*-*T*_*CYC1*_-*P*_*PGK1*_-*XYL2*-*T*_*CYC1*_-*P*_*ADH1*_-*XK*-*T*_*CYC1*_	[49]
pUGR-XYL2	pUG6, rDNA::*KanMX*-*P*_*PGK1*_-*XYL2*-*T*_*CYC1*_	This study
pRS41H	ARS/CEN plasmid with hygromycin B resistance gene for selection in yeast	EUROSCARF
pKT127	Optimized cassette for yEGFP tagging in *S. cerevisiae*	EUROSCARF
p416-UAS_*CLB*(3X)_-*P*_*TEF*_-lacZ	Plasmid containing a hybrid promoter based on *P*_*TEF*_ core with three tandem upstream activation sequences of *CLB2* promoter	[22]
pRS41H-yEGFP	Plasmid containing yEGFP open reading frame and ADH1 terminator	This study
pADH1	Reporter plasmid for *P*_*ADH1*_	This study
pADH2	Reporter plasmid for *P*_*ADH2*_	This study
pHSP12	Reporter plasmid for *P*_*HSP12*_	This study
pHSP26	Reporter plasmid for *P*_*HSP26*_	This study
pPGK1	Reporter plasmid for *P*_*PGK1*_	This study
pTDH3	Reporter plasmid for *P*_*TDH3*_	This study
pTEF1	Reporter plasmid for *P*_*TEF1*_	This study
pTPS1	Reporter plasmid for *P*_*TPS1*_	This study
p3xC-TEF1	Reporter plasmid for *P*_*3xC*-*TEF1*_	This study

**Table 2:** Strains used in this study

Strain	Description	Source or references
*Escherichia coli* strain		
*E. coli* DH5α	For plasmid construction and propagation	Invitrogen Ltd
*S. cerevisiae* strains		
S288c	Laboratory haploid strain, *MATα SUC2 gal2 mal2 mel flo1 flo8*-*1 hap1 ho bio1 bio6*	Gifted by Prof. Jens Nielsen, Chalmers University, Sweden
BY4741	Laboratory haploid strain, *Mata; his3Δ1 leu2Δ0 met15Δ0 ura3Δ0*	EUROSCARF http://www.bio.uni-frankfurt.de
CEN.PK113-5D	Laboratory haploid strain, *MATa*; *MAL2*-*8*^*c*^ *SUC2 ura3*-*52*	EUROSCARF
113-5DX	Xylose-assimilating strain based on CEN.PK113-5D, *YPRCdelta15::P*_*PGK1*_-*XYL1*-*T*_*CYC1*_-*P*_*PGK1*_-*XYL2*-*T*_*CYC1*_-*P*_*ADH1*_-*XKS1*-*T*_*CYC1*_	EUROSCARF
LX03	113-5D, *rDNA::KanMX*-*P*_*PGK1*_-*XYL2*-*T*_*CYC1*_	This study
BY-yEGFP	BY4741 with pRS41H-yEGFP	This study
BY-ADH1	BY4741 with reporter plasmid for *P*_*ADH1*_	This study
BY-ADH2	BY4741 with reporter plasmid for *P*_*ADH2*_	This study
BY-HSP12	BY4741 with reporter plasmid for *P*_*HSP12*_	This study
BY-HSP26	BY4741 with reporter plasmid for *P*_*HSP26*_	This study
BY-PGK1	BY4741 with reporter plasmid for *P*_*PGK1*_	This study
BY-TDH3	BY4741 with reporter plasmid for *P*_*TDH3*_	This study
BY-TEF1	BY4741 with reporter plasmid for *P*_*TEF1*_	This study
BY-TPS1	BY4741 with reporter plasmid for *P*_*TPS1*_	This study
BY-3xC-TEF1	BY4741 with reporter plasmid for *P*_*3xC*-*TEF1*_	This study
LX03-yEGFP	LX03 with pRS41H-yEGFP	This study
LX03-ADH1	LX03 with reporter plasmid for *P*_*ADH1*_	This study
LX03-ADH2	LX03 with reporter plasmid for *P*_*ADH2*_	This study
LX03-HSP12	LX03 with reporter plasmid for *P*_*HSP12*_	This study
LX03-HSP26	LX03 with reporter plasmid for *P*_*HSP26*_	This study
LX03-PGK1	LX03 with reporter plasmid for *P*_*PGK1*_	This study
LX03-TDH3	LX03 with reporter plasmid for *P*_*TDH3*_	This study
LX03-TEF1	LX03 with reporter plasmid for *P*_*TEF1*_	This study
LX03-TPS1	LX03 with reporter plasmid for *P*_*TPS1*_	This study
LX03-3xC-TEF1	LX03 with reporter plasmid for *P*_*3xC*-*TEF1*_	This study

### Promoter strengths under stressful conditions

To investigate the impact of stressful conditions on the responses of promoters, *S. cerevisiae* BY4741-derivative strains with various reporter plasmids for all the promoters were subjected to different stress conditions, including high temperature (39 °C), or in the presence of acetic acid (3.6 g/L) or furfural (1.0 g/L), respectively. As shown in Fig. 1b, c, when exposed to these stress conditions, longer lag phase of yeast cells was observed, and glucose were consumed at relatively lower rate, suggesting the inhibition of cell metabolism by different stressors.

**Fig. 2:**
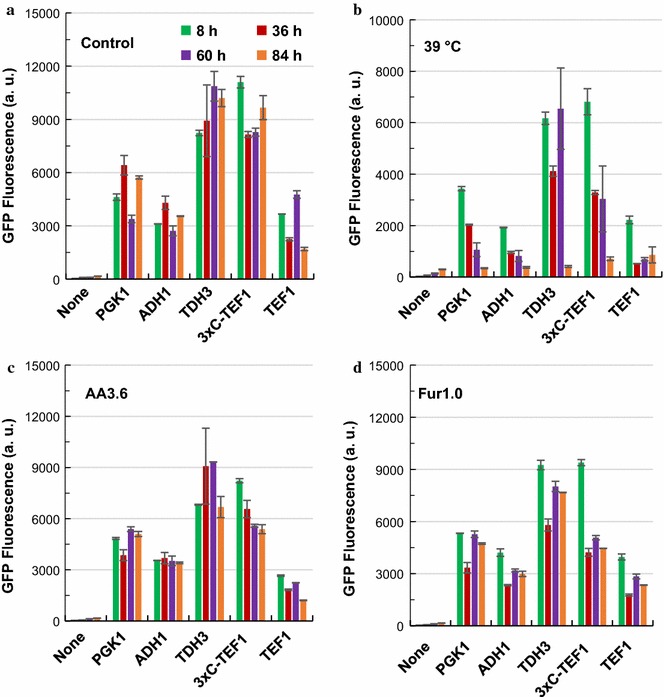
Time-course of the strengths of ‘constitutive promoters’ under stressful conditions. *S. cerevisiae* BY4741 derivative strains with different reporter plasmids were cultured in YPD100 medium under various stressful conditions. **a** Control condition without stress; **b** at 39 °C; **c** with 3.6 g/L acetic acid; **d** with 1.0 g/L furfural. The results were displayed in mean ± standard deviation

Strengths of constitutive promoters were first determined and compared in single yeast cells under stressful conditions. Among all the promoters, *P*_*3xC*-*TEF1*_ and *P*_*TDH3*_ were always the two strongest promoters under the stressful conditions tested, albeit the strength of *P*_*TDH3*_ was slightly higher than *P*_*3xC*-*TEF1*_ (Fig. 2). The strengths of the ‘constitutive’ promoters were relatively stable under the control condition as well as acetic acid and furfural treatments (Fig. 2c, d). In contrast, when cells were exposed to 39 °C, the strengths of all ‘constitutive’ promoters at 8 h was 60–75% of those corresponding values at 30 °C (Fig. 2b). In addition, the strengths of *P*_*ADH1*_ and *P*_*PGK1*_ decreased over the course of fermentation at 39 °C and resulted in only 10–20% of the initial strengths at 84 h (Fig. 2b). The decreased promoter activities at 39 °C correlated to strong growth inhibition under high temperature (Fig. 1b). However, relatively strong promoter activities were observed in the case of *P*_*TDH3*_ and *P*_*3xC*-*TEF1*_ even until 60 h. Compared with the native promoter *P*_*TEF1*_, the synthetic promoter *P*_*3xC*-*TEF1*_ showed better stability under high temperature (Fig. 2b). Similar to high temperature, acetic acid also exerted strong inhibitory effect to cell growth (Fig. 1b). Decreased strengths of *P*_*3xC*-*TEF1*_ and *P*_*TEF1*_ during ethanol fermentation were also observed in the presence of 3.6 g/L acetic acid, but not in the presence of 1.0 g/L furfural. Interestingly, although no apparent growth inhibition was observed by furfural addition (Fig. 1b), lower promoter activities were still observed in the five strong promoters we tested (Fig. 2d), implying inhibition of transcription or translation by furfural, which was revealed by the previous study [31].

**Fig. 3:**
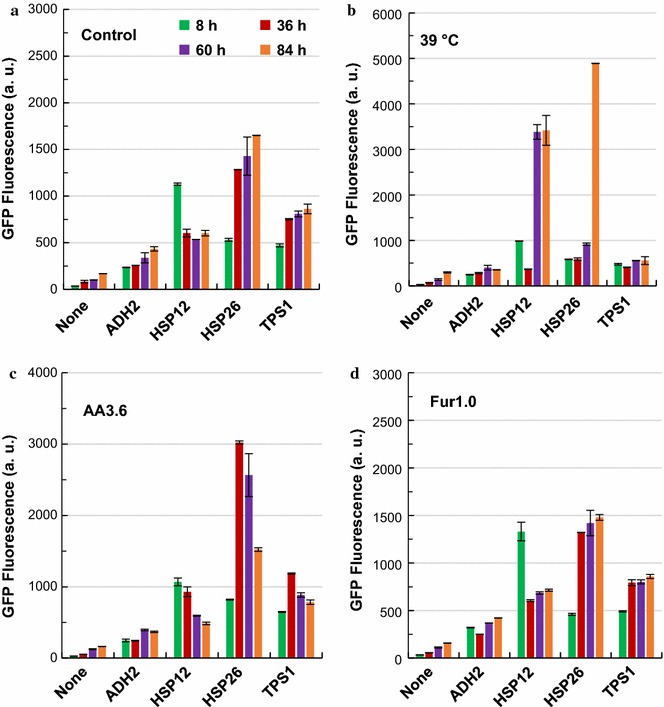
Time course of the strengths of ‘inducible promoters’ under stressful conditions. *S. cerevisiae* BY4741 derivative strains with corresponding reporter plasmids were cultured in YPD100 medium under various stressful conditions. **a** Control condition without stress; **b** at 39 °C; **c** with 3.6 g/L acetic acid; **d** with 1.0 g/L furfural. The results were displayed in mean ± standard deviation

In addition to the ‘constitutive’ promoters, four ‘inducible’ promoters, namely, *P*_*HSP12*_, *P*_*HSP26*_, *P*_*ADH2*_ and *P*_*TPS1*_ were also investigated. Compared with the ‘constitutive’ promoters, the ‘inducible’ promoters showed much lower strengths in all the conditions investigated (Fig. 3). When 1.0 g/L furfural was supplemented into the fermentation medium (YPD_100_), all these ‘inducible’ promoters showed similar dynamic pattern as that of the control (YPD_100_ without supplementation) (Fig. 3a, d). However, different patterns were observed in the case of acetic acid and high temperature conditions. Under acetic acid stress, both *P*_*HSP12*_ and *P*_*HSP26*_ showed elevated yEGFP expression levels, and *P*_*HSP26*_ exhibited the highest expression level at 36 h, which was more than five times of the control level without stress treatment (Fig. 3c). As the promoters of heat shock protein genes, *P*_*HSP12*_ and *P*_*HSP26*_ showed higher strengths when exposed to 39 than 30 °C (Fig. 3b), which is consistent with the previous report [32, 33]. Although the strengths of other promoters decreased significantly at 39 °C, yEGFP under the control of *P*_*HSP12*_ and *P*_*HSP26*_ displayed significantly increased expression levels at stationary phase (Fig. 3b).

### Promoter strengths in the presence of xylose

**Fig. 4:**
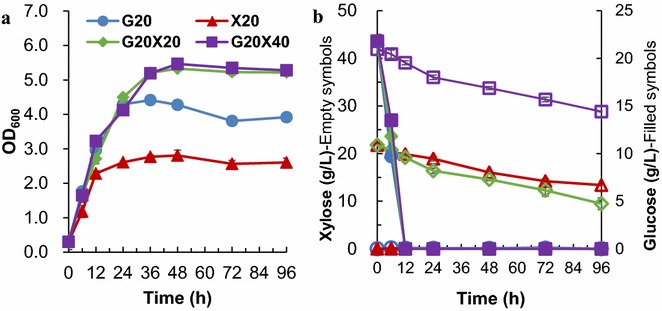
Cell growth (**a**) and consumption of glucose and xylose (**b**) with LX03 in the mixed sugar medium. *S. cerevisiae* LX03-derivative strains with the reporter plasmids were cultured at 30 °C and 200 rpm in the YP medium with different concentrations of glucose and xylose. G, glucose; X, xylose. Detailed information was presented in the main text. The results were displayed in mean ± standard deviation

Although growth of *S. cerevisiae* BY4741 was detected using xylose as the sole carbon source (Additional file 1: Figure S3), no significant consumption of xylose was detected (data not shown). Therefore, the recombinant strain *S. cerevisiae* LX03 harboring xylose utilization pathway was employed for the determination of promoter strengths in the presence of xylose. The promoter strengths in glucose-xylose mixture with different ratios were investigated. Cell growth of the yeast strains under these conditions was compared firstly (Fig. 4). Similar growth rates were observed in different medium (G20, G20X20 and G20X40), whereas much slower growth was observed when xylose was used as the sole carbon source (Fig. 4a). About 50% xylose was consumed when 20 g/L xylose was present, but slower xylose consumption rate was observed when 40 g/L xylose was supplemented (Fig. 4b). Due to the low inoculation size, xylose was not consumed completely, which enabled us to observe the promoter activities during xylose utilization.

**Fig. 5:**
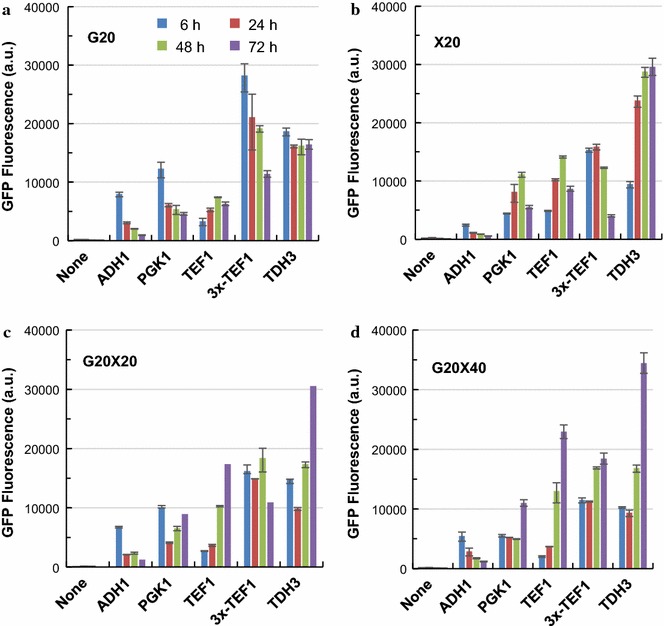
Time-course of promoter strengths of the ‘constitutive promoters’ in the presence of xylose. *S. cerevisiae* LX03-derivative strains with the reporter plasmids were cultured at 30 °C and 200 rpm in YP medium with different concentration of glucose and xylose. G, glucose; X, xylose; 20 and 40 after G or X represent the concentration (g/L) of glucose or xylose used. **a** G20; **b** X20; **c** G20X20; **d** G20X40. Detailed information was presented in the main text. The results were displayed in mean ± standard deviation

The responses of all the ‘constitutive’ promoters in the presence of xylose were subsequently compared in *S. cerevisiae* LX03 (Fig. 5). The promoter strength in descend order was *P*_*TDH3*_≈* P*_*3xC*-*TEF1*_> *P*_*PGK1*_≈* P*_*TEF1*_> *P*_*ADH1*_ when 20 g/L glucose was used as the sole carbon source (Fig. 5a). When comparing different time points, the strengths of *P*_*PGK1*_, *P*_*ADH1*_ and *P*_*3xC*-*TEF1*_ decreased significantly after glucose was depleted, but the activity of *P*_*TDH3*_ was relatively stable (Fig. 5a). When xylose was used as the sole carbon source, the highest promoter activity was observed in *P*_*TDH3*_, followed by *P*_*3xC*-*TEF1*_ and *P*_*TEF1*_ (Fig. 5b). Moderate promoter strength was displayed by *P*_*PGK1*_, and *P*_*ADH1*_ is the weakest constitutive promoter with xylose as the sole carbon source (Fig. 5b). The results suggested that *P*_*ADH1*_ may not be a good choice to drive gene expression in xylose. It is worth noting that, the initial strengths of *P*_*PGK1*_, *P*_*ADH1*_, *P*_*TDH3*_ and *P*_*3xC*-*TEF1*_ in yeast cells grown on medium with xylose as the sole carbon source were much lower than those grown on glucose (Fig. 5b). Similar results were observed when cells were cultured in mixed sugar (G20X20 and G20X40) conditions (Fig. 5c, d). When comparing *P*_*3xC*-*TEF1*_ with its native counterpart *P*_*TEF1*_, more stable promoter activities were observed in the presence of xylose (Fig. 5). The strength of native *P*_*TEF1*_ changes most significantly during mixed-sugar fermentation, and the highest activity at 72 h was almost 5 times of that at 6 h. However, the strength of *P*_*3xC*-*TEF1*_ only changed slightly throughout cultivation (Fig. 5c, d). We reasoned that the difference between the hybrid promoter and its native counterpart is mainly due to the addition of the tandem 240-bp UASs from the *CLB2* promoter (− 867 to − 627) [22, 34].

**Fig. 6:**
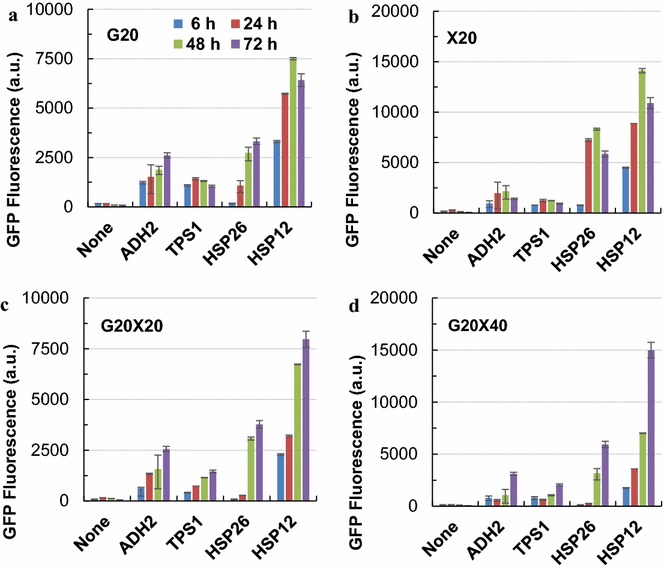
Time-course of promoter strengths of ‘inducible promoters’ in the presence of xylose. *S. cerevisiae* LX03-derivative strains carrying the reporter plasmids were cultured at 30 °C and 200 rpm in YP medium with different concentrations of glucose and xylose. G, glucose; X, xylose; 20 and 40 after G or X represent the concentration (g/L) of glucose or xylose used. **a** G20; **b** X20; **c** G20X20; **d** G20X40. Detailed information was presented in the main text. The results were displayed in mean ± standard deviation

The ‘inducible’ promoters showed different profiles with glucose as the sole carbon source, with *P*_*HSP26*_ and *P*_*ADH2*_ exhibited similar strength profiles, but the strengths of *P*_*HSP26*_ showed higher variety than that of *P*_*ADH2*_ (Fig. 6a). The results are consistent with those described in the literature [35], in which elevated protein expression level under the control of both *P*_*HSP12*_ and *P*_*HSP26*_ were observed under glucose starvation condition. In contrast to low promoter activities in glucose, higher yEGFP expression levels directed by *P*_*HSP12*_ and *P*_*HSP26*_ was observed when xylose was used as the sole carbon source (Fig. 6b). In addition, very low activities of *P*_*ADH2*_ and *P*_*TPS1*_ were observed in all the four conditions examined in this study. Stronger activities of *P*_*HSP12*_ comparing with that of *P*_*HSP26*_ were especially clear in the mixed sugar conditions (Fig. 6c, d).

## Discussion

Our current studies revealed dynamic changes of promoter strengths under different conditions, which provided useful information for developing yeast strains for practical applications. In the previous study, the promoter strengths of *P*_*ADH2*_, *P*_*TEF1*_, *P*_*PGK1*_, *P*_*TDH3*_ and *P*_*TPS1*_ in the presence of different carbon sources and across the diauxic shift were compared [2]. Recently, the activities of 29 promoters under aerobic or anaerobic xylose cultivation were evaluated [30], however, the responses of promoter strengths toward stressful condition were not investigated. Most reported studies focused on promoters of the central carbon metabolism genes [4, 5, 30]. The current studies are different from these studies in that inducible promoters such as *P*_*ADH2*_, *P*_*TPS1*_, *P*_*HSP12*_ and *P*_*HSP26*_ as well as the artificially synthesized promoter were investigated, and various stress conditions as well as xylose-utilization were focused. In combination with the previous studies, our studies provide useful information on optimizing gene expression for construction of yeast cell factories.

Generally, ‘constitutive promoters’ showed relatively higher promoter strengths toward various conditions compared to ‘inducible’ promoters. However, significant lower activities of *P*_*ADH1*_ and *P*_*PGK1*_ were also observed with xylose or galactose as the sole carbon source (Additional file 1: Figure S4). It seems that the high strengths of *P*_*ADH1*_ and *P*_*PGK1*_ depended on the presence of glucose. *P*_*ADH1*_ and *P*_*PGK1*_ were also sensitive to stressful conditions. These results revealed that the so-called ‘constitutive promoters’ also exhibit dynamic changes in their strengths under various stress conditions, and *P*_*ADH1*_ and *P*_*PGK1*_ might not be good choices for constantly high expression of genes under stressful conditions or when xylose is used as a carbon source. Dynamic activities were also observed in 37 promoters, including *P*_*ADH1*_, *P*_*ADH2*_, *P*_*TEF1*_, *P*_*PGK1*_, *P*_*TDH3*_ and *P*_*HSP26*_, characterized in three media (CSM, YPD and YPGal) [36]. In combination with the results on the dynamic promoter activities reported in this study, more rational control of gene expression can be achieved.

*P*_*HSP12*_ and *P*_*HSP26*_ exhibited higher strengths in the presence of xylose compared to those with glucose as the sole carbon source. It was reported that xylose concentration higher than 20 g/L may cause stress to the *S. cerevisiae* strains [37]. In addition, *P*_*HSP12*_ and *P*_*HSP26*_ exhibited high transcription level as well as high ribosome occupancy at glucose starvation condition [35]. Therefore, we speculated that the relatively high protein expression levels of yEGFP driven by *P*_*HSP12*_ and *P*_*HSP26*_ in the presence of xylose may be a combined consequence of glucose depletion and xylose stress, since these two promoters response to both glucose starvation and stressful conditions. Previously, two-stage transcriptional control of xylose assimilating pathway was achieved by employing *P*_*HSP26*_ to fine-tune the expression of *TAL1* and *XKS1*, which resulted in elevated xylose consumption rate and ethanol yield from xylose compared to traditional design with constitutive promoter *P*_*PGK1*_ [7]. However, so far no related study has been reported using *P*_*HSP12*_ to construct xylose-utilizing yeast. Our results revealed that *P*_*HSP12*_ can be used to develop xylose utilizing yeast strains.

In the current study, the strengths of the hybrid promoter under stress fermentation condition as well as in the presence of xylose were investigated. It seems that the hybrid promoter (*P*_*3xC*-*TEF1*_) based on *P*_*TEF1*_ showed the highest strength among all the promoters under almost all conditions and exhibited high stability (Additional file 1: Table S2). The strength of *P*_*3xC*-*TEF1*_ is comparable to that of *P*_*TDH3*_, which is the strongest constitutive promoter in *S. cerevisiae*. Consistent with the previous report [22], *P*_*3xC*-*TEF1*_ showed 2–4 times higher strength, when compared with the native promoter *P*_*TEF1*_, both under stressful conditions and in xylose fermentation conditions. Our results suggested that the synthetic promoter *P*_*3xC*-*TEF1*_ could be used for consistent high expression of individual genes under various stressful conditions.

According to the previous reports, promoter sequences can not only influence transcribed mRNA levels, but also the localization and ribosome occupancy of mRNA [35]. On the other hand, post-translational modifications were reported to exert regulation on the central carbon metabolism genes [38], which might cause differences in mRNA and protein levels. Considering that protein expression is more closely related to mRNA level, we focused on protein levels of the reporter gene to compare the promoter activities. A relatively stable yEGFP was used as the reporter in this study to determine the protein produced driven by different promoters. In the future, fluorescence protein with short half-life will be compared for the possibility of real-time detection [39].

We also compared our results with the transcriptomic data in the literature. Significant different expression levels of *TPS1*, *ADH1* and *ADH2* at different phases during glucose–xylose co-fermentation using *S. cerevisiae* MA-R4 and MA-B4 were also revealed by transcriptomic analysis. However, no significant changes were observed in *PGK1* [40]. In another study, significantly changed expression levels of *HSP26*, *ADH1* and *ADH2* were also reported with an engineered xylose-utilizing *S. cerevisiae* sun049 exposed to high temperature [41], which is consistent with our results. In contrast, among the corresponding ORFs of the eight native promoters studied, only *ADH1* showed slightly decreased transcription level during the ethanol fermentation in the presence of acetic acid or furfural-acetic acid mixture [31]. However, most of the transcriptomic studies were only performed with limited time points [31, 40, 41], which might underestimate the differences in different time points. Genetic background of the host strains and different culture conditions may also exert effects on the regulation of promoter activities. It was reported that growth rate acts as a determinant factor for gene expression level [42]. According to the previous report, the transcript levels of *ADH1* and *TEF1* were relatively stable at steady state with different growth rate [42], nevertheless, dynamic activities of *P*_*ADH1*_ and *P*_*TEF1*_ were observed either at high temperature (39 °C) or in the presence of xylose in this study. We assumed that the conditions employed in this study exert important influences on the promoter activities, and that these promoter activities are led by the combination of both environmental conditions and growth rate. The mechanisms for dynamic response of individual promoter need to be further investigated in future studies.

Previous study showed that the responses of the *P*_*CCW1*_ and *P*_*YGP1*_ toward low pH could be successfully changed by engineering the TFBSs in the promoter sequences [24], suggesting that TFBSs play critical role in the response of promoters. We therefore analyzed whether there is any correlation of TFBSs and the response of promoter strength. It was reported that Fkh1p/Fkh2p was critical for *CLB2* transcription under normal and oxidative stress conditions [43]. Compared with the endogenous promoter *P*_*TEF1*_, 12 more TFBSs for Fkh1p/Fkh2p were found in the synthetic promoter *P*_*3xC*-*TEF1*_ (Table 3), which may be responsible for its higher strengths under various conditions. More than four TFBSs of Haa1p, a transcription factor responsible for adaptation and tolerance to weak acids in *S. cerevisiae* [44, 45], were found in *P*_*HSP12*_ and *P*_*TPS1*_, which response to acetic acid stress. Both heat shock elements (HSEs) and stress response elements (STREs) are responsible for response to multiple stresses, and STREs are responsible for the high ribosome occupancy of mRNAs in cytoplasm in response to glucose starvation [35]. High numbers of STREs were found in inducible promoters *P*_*HSP12*_, *P*_*HSP26*_, and *P*_*TPS1*_, which was consistent with the increased strengths of these promoters in response stressful conditions as well as glucose depletion. Altogether, the predicted correlations of TFBSs and the response of promoter strength might shed lights on further optimization of synthetic promoters in future work.

**Table 3:** Transcription factor binding sites (TFBSs) involved in the promoter sequences

Counts	*P*_*PGK1*_	*P*_*ADH1*_	*P*_*TDH3*_	*P*_*ADH2*_	*P*_*TEF1*_	*P*_*HSP26*_	*P*_*HSP12*_	*P*_*TPS1*_	*P*_*3xC*-*TEF1*_	UAS_*CLB2*_
Hsf1p	3	1	1	1	1	1	0	1	1	0
Msn2p/Msn4p	1	2	3	0	2	*4*	*4*	*6*	1	0
Nrg1p	4	2	3	0	1	*5*	*5*	*9*	1	0
Gis1p/Rph1p	1	2	3	1	1	*4*	*4*	*6*	1	0
Haa1p	0	2	1	1	2	1	*4*	*5*	2	0
Yap1p	3	1	2	0	1	0	0	3	3	1
Stb5p	1	3	2	3	5	5	3	6	12	2
Crz1p	0	0	1	0	0	0	1	2	3	1
Rtg1p/Rtg3p	5	1	0	0	1	2	0	2	1	0
Rgt1p	1	1	0	1	0	1	0	1	0	0
Adr1p	0	0	0	*2*	0	*1*	0	0	0	0
Gcr1p	*6*	*6*	2	1	2	2	2	1	*6*	1
Azf1p	2	3	0	0	4	0	0	0	4	0
Gln3p	2	0	0	1	1	0	0	0	7	2
Mcm1p	1	0	1	0	0	0	0	0	*3*	*1*
Fkh1p/Fkh2p	3	2	*7*	2	0	3	3	2	*12*	*4*

Italic values indicate the numbers of putative TFBSs that are significantly larger than others

## Conclusions

*P*_*TDH3*_ and the synthetic hybrid promoter (*P*_*3xC*-*TEF1*_) showed the highest strength and stability in almost all conditions tested in this study, suggesting that synthetic promoter has the potential to achieve not only stronger activity, but also more stable expression under various time points and various conditions. The constitutive promoters exhibit dynamic changes in their strengths under various stress conditions. Among the inducible promoters, *P*_*HSP12*_ was superior in higher temperature and acetic acid stress, and showed the highest expression levels when xylose was used as the carbon source. Our results provide novel insights in promoter activities for further optimization of gene expression in practical applications.

## Methods

### Strains and plasmids propagation

The strains and plasmids used in this study were listed in Tables 1 and 2. *S. cerevisiae* strains were cultured in YP medium (containing 10 g/L yeast extract and 20 g/L peptone) supplemented with different carbon sources. Genomic DNA extracted from the model *S. cerevisiae* strain S288c (gifted by Prof. Jens Nielsen, Chalmers University, Sweden) was used for the amplification of native promoters. Laboratory strain *S. cerevisiae* BY4741 was used as the host for the determination of promoter strengths. To investigate the response of promoters in presence of xylose, a xylose assimilating strain *S. cerevisiae* LX03 containing xylose reductase-xylitol dehydrogenase (XR-XDH) pathway was used.

*Escherichia coli* DH5alpha was used for the propagation of plasmid and cultivated in Luria–Bertani medium (5 g/L yeast extract, 10 g/L tryptone and 10 g/L NaCl), 100 mg/L ampicillin was added for the selection of transformants.

### Construction of reporter plasmids and corresponding yeast strains

A set of reporter plasmids were constructed with yEGFP as the reporter (Table 1 and Additional file 1: Figure S1). The reporter plasmids are based on the ARS/CEN plasmid pRS41H with hygromycin B resistance gene as the selection marker. Eight native promoters, namely, *P*_*ADH1*_, *P*_*PGK1*_, *P*_*TEF1*_, *P*_*TDH3*_, *P*_*TPS1*_, *P*_*HSP12*_, *P*_*HSP26*_ and *P*_*ADH2*_, were included (Table 1). Among these promoters, *P*_*ADH1*_, *P*_*PGK1*_, *P*_*TEF1*_ and *P*_*TDH3*_ are commonly recognized as ‘constitutive’ promoters, while *P*_*TPS1*_, *P*_*HSP12*_, *P*_*HSP26*_ and *P*_*ADH2*_ are regarded as ‘inducible’ promoters. In addition, a hybrid promoter based on *P*_*TEF1*_ core with three tandem UASs of *CLB2* promoter [22] was also investigated, which was designated as the artificial promoter, *P*_*3xC*-*TEF1*_, in this study. The primers for the construction of reporter plasmid were listed in Additional file 1: Table S1. Optimized cassette for yEGFP containing yEGFP open reading frame (ORF) and *ADH1* (encoding alcohol dehydrogenase) terminator was amplified from pKT127 by polymerase chain reaction (PCR) with primers yEGFP-F/R, the PCR products were cloned into the multi-cloning sites (MCSs) of pRS41H plasmid between *Sma*I and *Kpn*I. The newly constructed plasmid was named as pRS41H-yEGFP. Then the sequences of eight native promoters were amplified by PCR with the genomic DNA of S288c as template and cloned into the MCSs of pRS41H-yEGFP within *Bam*HI and *Hin*dIII restriction site. The hybrid promoter (*P*_*3xC*-*TEF1*_, short for *UAS*_*CLB*(3X)_-*P*_*TEF*_) containing *P*_*TEF1*_ core with three tandem UASs of *CLB2* promoter (UAS_*CLB*_, 240 bp) was amplified by PCR from the plasmid p416-*UAS*_*CLB*(3X)_-*P*_*TEF*_-*lacZ* [22]. After digestion with the corresponding restrict enzymes, the promoter fragment was ligated into the plasmid. The diagram of the reporter plasmid was shown in Fig. 1 and the corresponding plasmid was listed in Table 1. After verification by sequencing, the plasmid was transformed into *S. cerevisiae* BY4741 or LX03 via LiAc/ssDNA/PEG method [46], the transformants were selected on YPD plates with 200 mg/L hygromycin B. Authentic transformants were obtained, and related information was listed in Table 2.

### Cell cultivation

To test promoter strengths in a high-throughput manner, the yeast cells harbor promoter-yEGFP expression cassette were cultivated in 24 deep-well baffled plates (Catalog: YD010124B, Changzhou Yingde Bio-Technology Co., Ltd, Jiangsu, China). Firstly, yeast cells stored in 15% (v/v) glycerol at − 80 °C were inoculated in 100 mL shake flask containing 20 mL YPD medium with 200 mg/L hygromycin B and cultivated at 30 °C, 150 rpm for 16 h. Then 200 μL of the culture was transferred into the 24 deep-well baffled plates with 3 mL growth or fermentation medium with 200 mg/L hygromycin B. The cells were cultivated at 200 rpm, and 39 °C was used to examine the response of promoters toward high temperature, otherwise the cells were cultivated at 30 °C. To test the response of promoters toward different conditions, yeast cells were cultured in different media. Cell growth and promoter strength were compared in BY4741 background under different carbon sources, including 20 g/L glucose (G20), 20 g/L xylose (X20), 20 g/L galactose (Gal20), 20 g/L glycerol (Gly20) and a mixture of 20 g/L glucose + 10 g/L xylose (G20X10). Besides, 40 g/L ethanol (Eth40) and 5.0 g/L (AA5.0) acetic acid were supplemented into YPD to investigate the response under the stress of ethanol and acetic acid, respectively.

*S. cerevisiae* BY4741 derivative strains were used to determine the promoter strength throughout the ethanol fermentation process, with or without inhibition factors. Cells were cultured in YPD100 (10 g/L yeast extract, 20 g/L peptone and 100 g/L glucose) medium under the following conditions: (1) 30 °C and 200 rpm (control); (2) 39 °C and 200 rpm (39 °C); (3) 30 °C, 200 rpm and in the presence of 3.6 g/L acetic acid (AA3.6); (4) 30 °C, 200 rpm and in the presence of 1.0 g/L furfural (Fur1.0).

The responses of promoter strengths in presence of xylose were compared in the xylose assimilating *S. cerevisiae* LX03. Cells were cultured at 30 °C and 200 rpm in YP medium supplemented with the following carbon sources: 20 g/L glucose (G20); 20 g/L xylose (X20); 20 g/L glucose and 20 g/L xylose (G20X20); and 20 g/L glucose and 40 g/L xylose (G20X40). The cell growth and promoter strengths throughout the co-fermentation of glucose and xylose were determined.

### Determination of cell density

The cell density in the culture plates was determined by measuring optical density at 600 nm (OD_600_) via spectrophotometer (Thermo-Fisher Scientific™, Multiskan™ GO, MA, USA) with the 96-well plate. Samples were withdrawn from the cell culture, diluted with ddH_2_O to an OD_600_ within 0.2–0.8, 200 μL of the diluted suspension was added into the plates for analysis.

### Determination of promoter strengths by flow cytometry

The determination of promoter strengths was referred to the method previously described by Peng et al. [2]. GFP fluorescence in single cells was analyzed, immediately after sampling, using a flow cytometer (BD FACSAria™ II, BD Biosciences, USA). GFP fluorescence was excited by a 488 nm laser and monitored through a FL1. A filter (wavelength 530/20 nm). 10,000 or 50,000 events were counted for each sample to get convincible results. The particle volume and complexity for each event were monitored by forward scatter detector (FSC.A) and side scatter detector (SSC.A).

### Correlation of mRNA level and promoter strength

The correlation of mRNA levels and promoter strengths selected strains harbor different promoters were determined with their corresponding mRNA levels and promoter strengths. The method for determination of the promoter strengths was as described above. The mRNA levels were determined by real-time quantitative PCR (RT-qPCR). Total RNA were extracted with plant total RNA extraction kit (Sangon, Shanghai). After reverse transcription with the reverse transcription kit (Takara Ltd, Dalian, China), the mRNA level was determined by real-time RT-PCR kit (Bio-Rad). The mRNA levels of *yEGFP* in different strains were determined with *ALG9* as the internal control gene [47], and the primers for detection of *ALG9* and *yEGFP* were listed in Additional file 1: Table S1.

### Prediction of TFBSs in promoter sequences

The TFBSs in the sequences individual promoter as well as in UAS_*CLB2*_ was predicted on YEASTACT database (http://www.yeastract.com/) following the previously described method [48].

### HPLC analysis

The concentration of glucose, xylose, ethanol, acetate, glycerol and other components in fermentation broth were analyzed by high performance liquid chromatography system (HPLC, Waters e2695, Waters, MA, USA) equipped with a Refractive Index Detector (RI, Waters 2414, Waters, MA, USA) and Aminex HPX-87H column (300 mm × 7.8 mm, Bio-Rad, Hercules, CA). The operating temperature of the column and RI were 65 and 50 °C, respectively, and 4 mmol/L H_2_SO_4_ was used as the mobile phase. All the samples withdrew from the fermentation broth were centrifuged at 10,000×*g* at room temperature for 2 min, the supernatants were then diluted and filtrated with 0.22 μm filter before sampling. Twenty microliter of diluted samples were injected into the HPLC system for analysis.

### Statistical analysis

All the experiments were duplicated and the results were expressed as mean value and standard derivations.

## Additional file

**Additional file 1: Table S1.** Primers used in this study. **Table S2.** Ranking of promoter strengths at log phase under various conditions. **Table S3.** Sequence analysis of the promoters. **Table S4.** Predicted transcription factor (TF) binding sites involved in the promoter sequences. **Figure S1.** The GFP fluorescence of yeast cells under the control of various promoters and in the presence or absence of hygromycin B. **Figure S2.** Correlation of yEGFP fluorescence and mRNA levels. **Figure S3.** Cell growth of *S. cerevisiae* BY4741 under various conditions. **Figure S4.** The promoter strengths in log-phase cells under different conditions.

## Abbreviations

*P*_*XXXN*_: the promoter of the gene, *XXXN*
GFP: green fluorescence protein
yEGFP: yeast enhanced green fluorescence protein
UAS: upstream activation sequences
TFBS: transcription factor binding sites
