# Supplementary material for: Condition-specific promoter activities in Saccharomyces cerevisiae
Source: Microb Cell Fact. 2018 Apr 10;17:58. doi: 10.1186/s12934-018-0899-6 (PMC5891911; doi:10.1186/s12934-018-0899-6)
Supplement: Supplementary file 1 — Additional file 1: Table S1. Primers used in this study. Table S2. Ranking of promoter strengths at log phase under various conditions. Table S3. Sequence analysis of the promoters. Table S4. Predicted transcription factor (TF) binding sites involved in the promoter sequences. Figure S1. The GFP fluorescence of yeast cells under the control of various promoters and in the presence or absence of hygromycin B. Figure S2. Correlation of yEGFP fluorescence and mRNA levels. Figure S3. Cell growth of S. cerevisiae BY4741 under various conditions. Figure S4. The promoter strengths in log-phase cells under different conditions. [file 12934_2018_899_MOESM1_ESM.docx]

Supplementary Information for

# Condition-specific promoter activities in *Saccharomyces cerevisiae*

Liang Xiong^1^, Yu Zeng^2^, Ruiqi Tang^2^, Hal S. Alper^3^, Fengwu Bai^2^, Xinqing Zhao^2^*

^1^School of Life Science and Biotechnology, Dalian University of Technology, Dalian 116024, China.

^2^State Key Laboratory of Microbial Metabolism (SKLMM), School of Life Science and Biotechnology, Shanghai Jiao Tong University, Shanghai 200240, China.

^3^Department of Chemical Engineering, The University of Texas at Austin, Austin, Texas 78712, USA

*Corresponding author: Xinqing Zhao

Address: School of Life Science and Biotechnology, Shanghai Jiao Tong University, Shanghai 200240, China.

Tel/Fax: +86-21-34206673/+86-21-34208028

E-mail: xqzhao@sjtu.edu.cn

**Supplementary Tables**

Table S1. Primers used in this study.

Table S2. Ranking of promoter strengths at log phase under various conditions.

Table S3. Sequence analysis of the promoters.

Table S4. Predicted transcription factor (TF) binding sites involved in the promoter sequences.

**Table S1. Primers for construction of reporter plasmids and RT-qPCR**

| Primer | Sequence* （5’ →3’） |
| --- | --- |
| yEGFP-F | TCCCCCCGGGGTCGACGGTGACGGTGCTGGTTTAA |
| yEGFP-R | GGAGGTACCCCTGTTATCCCTAGCGGATCT |
| ADH1p-F | TCCGGATCCAGGGGGATCGAAGAAATGATG |
| ADH1p-R | TGCAAGCTTTGTATATGAGATAGTTGATTG |
| ADH2p-F | TCCGGATCCTCTCTCCGGTTACAGCCTG |
| ADH2p-R | TGCAAGCTTTGTGTATTACGATATAGTTAATAG |
| PGK1p-F | TCCGGATCCGGAAGTACCTTCAAAGAATG |
| PGK1p-R | TGCAAGCTTTTGTTTTATATTTGTTGTAAAAAG |
| TEF1p-F | TCCGGATCCGCACACACCATAGCTTCAAA |
| TEF1p-R | TGCAAGCTTTTGTAATTAAAACTTAGATTAG |
| TDH3p-F | TCCGGATCCCAGTTTATCATTATCAATACTGCC |
| TDH3p-R | TGCAAGCTTGAATCCGTCGAAACTAAGTTCTGGTG |
| 3xC-TEF1-F | CGCGGATCCCACTAAAGGGAACAAAAGCTGG |
| 3xC-TEF1-R | ACGCGTCGACAAACTTAGATTAGATTGCTA |
| TPS1p-F | TCCGGATCCCAACCCGGTCTCGAAGAACA |
| TPS1p-R | TGCAAGCTTAGTTCTATGTCTTAATAAGTC |
| HSP12p-F | TCCGGATCCAACTTGCAGGGGGCGGCAC |
| HSP12p-R | TGCAAGCTTTGTTGTATTTAGTTTTTTTTGTTTTGAG |
| HSP26p-F | TCCGGATCCTTTGGCTCCTTGAAAACAAG |
| HSP26p-R | TGCAAGCTTGTTAATTTGTTTAGTTTGTT |
| rtALG9 | ATCGTGAAATTGCAGGCAGCTTGG |
| rtALG9 | CATGGCAACGGCAGAAGGCAATAA |
| rtyEGFP | ATGGCTGACAAACAAAAGAATG |
| rtyEGFP | CAGATTGAGTGGATAAGTAATG |

*The underlined letters was the sequences for the restriction sites used.

**Table S2. Ranking of promoter strengths at log phase under various conditions**

| **Strains** | | **BY4741** | | | | | | | | | | **LX03** | | | | **Sum** |
| --- | --- | --- | --- | --- | --- | --- | --- | --- | --- | --- | --- | --- | --- | --- | --- | --- |
| **Promoters** | System ID | **G20** | **X20** | **G20X10** | **Gal20** | **Eth40** | **AA5.0** | **G100** | **39 ^o^C** | **AA3.6** | **Fur1.0** | **G20** | **X20** | **G20X20** | **G20X40** |  |
| ***ADH2*** | *YMR303C* | **1** | **1** | **1** | **1** | **1** | **1** | **1** | **1** | **1** | **1** | **3** | **3** | **3** | **2** | **21** |
| ***TPS1*** | *YBR072W* | **2** | **2** | **3** | **2** | **3** | **2** | **2** | **2** | **2** | **3** | **2** | **2** | **2** | **3** | **32** |
| ***HSP26*** | *YBR126C* | **3** | **7** | **2** | **3** | **4** | **7** | **3** | **3** | **3** | **2** | **1** | **1** | **1** | **1** | **41** |
| ***HSP12*** | *YPR080W* | **4** | **3** | **4** | **5** | **5** | **3** | **4** | **4** | **4** | **4** | **5** | **6** | **4** | **4** | **59** |
| ***ADH1*** | *YCR012W* | **5** | **4** | **6** | **7** | **6** | **6** | **5** | **5** | **6** | **6** | **6** | **4** | **6** | **6** | **78** |
| ***TEF1*** | *YOL086C* | **6** | **6** | **5** | **6** | **7** | **5** | **6** | **6** | **5** | **5** | **4** | **7** | **5** | **5** | **78** |
| ***PGK1*** | *YFL014W* | **7** | **5** | **7** | **4** | **2** | **4** | **7** | **7** | **7** | **7** | **7** | **5** | **7** | **7** | **83** |
| ***TDH3*** | *YGR192C* | **8** | **8** | **9** | **8** | **8** | **8** | **8** | **8** | **8** | **8** | **8** | **8** | **8** | **8** | **113** |
| ***3xC-TEF1*** | *YOL086C** | **9** | **9** | **8** | **9** | **9** | **9** | **9** | **9** | **9** | **9** | **9** | **9** | **9** | **9** | **125** |

The strengths of nine promoters under each condition were ranked from 1 to 9 and were expressed with different colors.

**Conditions:** 10 g/L Yeast extract, 20 g/L peptone plus (+), G20, 20 g/L glucose; X20, 20 g/L xylose; G20X10, 20 g/L glucose and 10 g/L xylose; Gal20, 20 g/L galactose; Eth40, 20 g/L glucose and 40 g/L ethanol; AA5.0, 20 g/L glucose and 5.0 g/L acetic acid; G100, 100 g/L glucose; 39˚C, 100 g/L glucose and 39 ^o^C; AA3.6, 100 g/L glucose and 3.6 g/L acetic acid; Fur1.0, 100 g/L glucose and 1.0 g/L furfural; G20X20, 20 g/L glucose and 20 g/L xylose; G20X40, 20 g/L glucose and 40 g/L xylose.

**Table S3. Sequence analysis of the promoters.**

| Promoters | Lengths (bp) | GC Content (%) | TATA box and its location |
| --- | --- | --- | --- |
| *P_PGK1_* | 981 | 37.07 | TATATAAA (-151 ~ -144)  or TATATATA (-153 ~ -146) |
| *P_ADH1_* | 759 | 38.87 | TATAAATA (-128 ~ -121) |
| *P_TDH3_* | 673 | 35.36 | TATATAAA (-141 ~ -134) |
| *P_ADH2_* | 475 | 36.84 | TATAAATA (-161 ~ -154) |
| *P_TEF1_* | 420 | 33.57 | TTAATAAA (-122 ~ -115) |
| *P_HSP26_* | 699 | 40.49 | TATAAATA (-154 ~ -147) |
| *P_HSP12_* | 422 | 36.97 | TATAAATA (-145 ~ -138) |
| *P_TPS1_* | 602 | 49.08 | TATATATA (-178 ~ -171) |
| *P_3xC-TEF1_* | 1147 | 36.88 | TTAATAAA (-118 ~ -111) |

**Table S4. Predicted transcription factor (TF) binding sites in the promoter sequences**

| TFs | Brief description | DNA binding sites* | References |
| --- | --- | --- | --- |
| Hsf1p | Trimeric heat shock transcription factor, activates multiple genes in response to stresses that include hyperthermia. | NGAANNTTCN NTTCNNGAAN | [[1](#_ENREF_1)] |
| Msn2p / Msn4p | Transcriptional activator activated in stress conditions, inducing gene expression. | CCCCT RGGGG AGGGG | [[2](#_ENREF_2)] |
| Haa1p | Transcriptional activator involved in adaptation and response to weak acid stress. | SMGGSG GAGGCG | [[3](#_ENREF_3), [4](#_ENREF_4)] |
| Nrg1p | Transcriptional repressor mediates glucose repression and negatively regulates a variety of processes including filamentous growth and alkaline pH response. | CCCCT CCCTC GGACCCT | [[5](#_ENREF_5), [6](#_ENREF_6)] |
| Gis1p / Rph1p | Histone demethylase and transcription factor involved in expression of genes during nutrient limitation. | TWAGGGAT CCCCT AGGGG | [[7](#_ENREF_7), [8](#_ENREF_8)] |
| Yap1p | Transcription factor required for oxidative stress tolerance; activated by H_2_O_2_ and mediates resistance to cadmium. | TGACTAA TKACAAA TGACAA | [[9](#_ENREF_9), [10](#_ENREF_10)] |
| Stb5p | Transcription factor, involved in regulating multidrug resistance and oxidative stress response. | TCTCCGCGAAC CGGNS | [[11](#_ENREF_11), [12](#_ENREF_12)] |
| Crz1p | Transcription factor, activates transcription of stress response genes | GGGGCTG GAGCCC | [[13](#_ENREF_13)] |
| Rtg1p / Rtg3p | Transcription factors that activate the retrograde (RTG) and TOR pathways. | GTCAC GGTAC | [[14](#_ENREF_14), [15](#_ENREF_15)] |
| Rgt1p | Glucose-responsive transcription factor that regulates expression of genes in response to glucose. | CGGANNA | [[16](#_ENREF_16), [17](#_ENREF_17)] |
| Adr1p | Carbon source-responsive transcription factor, required for transcription of the glucose-repressed genes. | TTGGRG | [[18](#_ENREF_18)] |
| Gcr1p | Transcriptional activator of genes involved in glycolysis. | CTTCC CWTCC | [[19-21](#_ENREF_19)] |
| Azf1p | Zinc-finger transcription factor involved in diauxic shift. | AAGAAAAA AAAAGAAA | [[22](#_ENREF_22), [23](#_ENREF_23)] |
| Gln3p | Transcriptional activator of genes regulated by nitrogen catabolite repression (NCR). | GATAAG GATTAG | [[24](#_ENREF_24), [25](#_ENREF_25)] |
| Mcm1p | Transcription factor involved in cell-type-specific transcription and pheromone response; plays a central role in the formation of both repressor and activator complexes. | DCCYWWWNNRG  CCYWWWNNRG | [[26](#_ENREF_26), [27](#_ENREF_27)] |
| Fkh1p / Fkh2p | Forkhead family transcription factor with a minor role in the expression of G2/M phase genes | [GTMAACAA](http://www.yeastract.com/view.php?existing=consensus&proteinname=Fkh1p&consensus=GTMAACAA) [RTAAAYAA](http://www.yeastract.com/view.php?existing=consensus&proteinname=Fkh1p&consensus=RTAAAYAA) [RYMAAYA](http://www.yeastract.com/view.php?existing=consensus&proteinname=Fkh1p&consensus=RYMAAYA) [RYAAACAWW](http://www.yeastract.com/view.php?existing=consensus&proteinname=Fkh1p&consensus=RYAAACAWW) | [[28](#_ENREF_28), [29](#_ENREF_29)] |

* Only the conserved sequences found in the promoter sequences were shown as the DNA binding sites of the transcription factors. The information of TF binding sites was obtained from YEATRACT [[30](#_ENREF_30)] (<http://www.yeastract.com/> ).

**Supplementary Figures**

**Figure S1. The yEGFP fluorescence of yeast cells under the control of various promoters and in the presence or absence of hygromycin.**

The GFP fluorescence of cells under the control of various promoters was detected in the presence or absence of hygromycin. The detailed methods were presented in the main text.

**Figure S2. Correlation of yEGFP fluorescence and mRNA levels.**

The yEGFP fluorescence and mRNA levels in cells grown in YPD with five different promoters were determined and analyzed. The detailed methods were presented in the main text.

**Figure S3. Cell growth of BY4741 under various conditions.**

*S. cerevisiae* BY4741 was grown in different YPD media (10 g/L Yeast Extract, 20 g/L Peptone) containing different carbon sources and inhibitors: (G20: 20 g/L Glucose; X20: 20 g/L Xylose; Gal 20: 20 g/L Galactose; Eth40: 20 g/L Glucose +40 g/L Ethanol; AA5.0: 20 g/L Glucose +5.0 g/L Acetic acid). The results showed were the mean and standard derivation of 6 parallel experiments.

**Figure S4. The promoter strengths in log-phase cells under different conditions.**

*S. cerevisiae* BY4741 was grown in different YPD media (10 g/L Yeast Extract, 20 g/L Peptone) containing different carbon sources and inhibitors (G20: 20 g/L Glucose; X20: 20 g/L Xylose; Gal 20: 20 g/L Galactose; Eth40: 20 g/L Glucose +40 g/L Ethanol; AA5.0: 20 g/L Glucose +5.0 g/L Acetic acid). Yeast cells were collected at log phase (6 h), and the promoter strengths were determined. The results showed was the mean and standard derivation of 3 parallel experiments.

**Figure S1.**

**Figure S2.**

**Figure S3.**

**Figure S4.**

# References:

1. Santoro N, Johansson N, Thiele DJ: **Heat shock element architecture is an important determinant in the temperature and transactivation domain requirements for heat shock transcription factor.** *Mol Cell Biol* 1998, **18:**6340-6352.

2. Martinez-Pastor MT, Marchler G, Schüller C, Marchler-Bauer A, Ruis H, Estruch F: **The Saccharomyces cerevisiae zinc finger proteins Msn2p and Msn4p are required for transcriptional induction through the stress response element (STRE).** *EMBO J* 1996, **15:**2227.

3. Mira NP, Henriques SF, Keller G, Teixeira MC, Matos RG, Arraiano CM, Winge DR, Sa-Correia I: **Identification of a DNA-binding site for the transcription factor Haa1, required for *Saccharomyces cerevisiae* response to acetic acid stress.** *Nucleic Acids Res* 2011, **39:**6896-6907.

4. Fernandes AR, Mira NP, Vargas RC, Canelhas I, Sa-Correia I: ***Saccharomyces cerevisiae* adaptation to weak acids involves the transcription factor Haa1p and Haa1p-regulated genes.** *Biochem Biophys Res Commun* 2005, **337:**95-103.

5. Lee SB, Kang HS, Kim T: **Nrg1 functions as a global transcriptional repressor of glucose-repressed genes through its direct binding to the specific promoter regions.** *Biochem Biophys Res Commun* 2013, **439:**501-505.

6. Vyas VK, Berkey CD, Miyao T, Carlson M: **Repressors Nrg1 and Nrg2 regulate a set of stress-responsive genes in Saccharomyces cerevisiae.** *Eukaryot Cell* 2005, **4:**1882-1891.

7. Zhang N, Wu J, Oliver SG: **Gis1 is required for transcriptional reprogramming of carbon metabolism and the stress response during transition into stationary phase in yeast.** *Microbiology* 2009, **155:**1690-1698.

8. Bernard A, Klionsky DJ: **Rph1 mediates the nutrient-limitation signaling pathway leading to transcriptional activation of autophagy.** *Autophagy* 2015, **11:**718-719.

9. Gounalaki N, Thireos G: **Yap1p, a yeast transcriptional activator that mediates multidrug resistance, regulates the metabolic stress response.** *EMBO J* 1994, **13:**4036.

10. Delaunay A, Isnard AD, Toledano MB: **H2O2 sensing through oxidation of the Yap1 transcription factor.** *EMBO J* 2000, **19:**5157-5166.

11. Akache B, Turcotte B: **New regulators of drug sensitivity in the family of yeast zinc cluster proteins.** *J Biol Chem* 2002, **277:**21254-21260.

12. Larochelle M, Drouin S, Robert F, Turcotte B: **Oxidative stress-activated zinc cluster protein Stb5 has dual activator/repressor functions required for pentose phosphate pathway regulation and NADPH production.** *Mol Cell Biol* 2006, **26:**6690-6701.

13. Yoshimoto H, Saltsman K, Gasch AP, Li HX, Ogawa N, Botstein D, Brown PO, Cyert MS: **Genome-wide analysis of gene expression regulated by the calcineurin/Crz1p signaling pathway in *Saccharomyces cerevisiae*.** *J Biol Chem* 2002, **277:**31079-31088.

14. Hashim Z, Mukai Y, Bamba T, Fukusaki E: **Metabolic profiling of retrograde pathway transcription factors rtg1 and rtg3 knockout yeast.** *Metabolites* 2014, **4:**580-598.

15. Rothermel BA, Thornton JL, Butow RA: **Rtg3p, a basic helix-loop-helix/leucine zipper protein that functions in mitochondrial-induced changes in gene expression, contains independent activation domains.** *J Biol Chem* 1997, **272:**19801-19807.

16. Ozcan S, Leong T, Johnston M: **Rgt1p of Saccharomyces cerevisiae, a key regulator of glucose-induced genes, is both an activator and a repressor of transcription.** *Mol Cell Biol* 1996, **16:**6419-6426.

17. Kim JH, Polish J, Johnston M: **Specificity and regulation of DNA binding by the yeast glucose transporter gene repressor Rgt1.** *Mol Cell Biol* 2003, **23:**5208-5216.

18. Biddick RK, Law GL, Young ET: **Adr1 and Cat8 mediate coactivator recruitment and chromatin remodeling at glucose-regulated genes.** *PLoS One* 2008, **3:**e1436.

19. Willis KA, Barbara KE, Menon BB, Moffat J, Andrews B, Santangelo GM: **The global transcriptional activator of *Saccharomyces cerevisiae*, Gcr1p, mediates the response to glucose by stimulating protein synthesis and CLN-dependent cell cycle progression.** *Genetics* 2003, **165:**1017-1029.

20. Uemura H, Koshio M, Inoue Y, Lopez MC, Baker HV: **The role of Gcr1p in the transcriptional activation of glycolytic genes in yeast *Saccharomyces cerevisiae*.** *Genetics* 1997, **147:**521-532.

21. Barbara KE, Haley TM, Willis KA, Santangelo GM: **The transcription factor Gcr1 stimulates cell growth by participating in nutrient-responsive gene expression on a global level.** *Mol Genet Genomics* 2006, **277:**171-188.

22. Stein T, Kricke J, Becher D, Lisowsky T: **Azf1p is a nuclear-localized zinc-finger protein that is preferentially expressed under non-fermentative growth conditions in *Saccharomyces cerevisiae*.** *Curr Genet* 1998, **34:**287-296.

23. Slattery MG, Liko D, Heideman W: **The function and properties of the Azf1 transcriptional regulator change with growth conditions in *Saccharomyces cerevisiae*.** *Eukaryot Cell* 2006, **5:**313-320.

24. Blinder D, Magasanik B: **Recognition of nitrogen-responsive upstream activation sequences of *Saccharomyces cerevisiae* by the product of the GLN3 gene.** *J Bacteriol* 1995, **177:**4190-4193.

25. Cox KH, Tate JJ, Cooper TG: **Cytoplasmic compartmentation of Gln3 during nitrogen catabolite repression and the mechanism of its nuclear localization during carbon starvation in *Saccharomyces cerevisiae*.** *J Biol Chem* 2002, **277:**37559-37566.

26. Althoefer H, Schleiffer A, Wassmann K, Nordheim A, Ammerer G: **Mcm1 is required to coordinate G2-specific transcription in *Saccharomyces cerevisiae*.** *Mol Cell Biol* 1995, **15:**5917-5928.

27. Acton TB, Zhong H, Vershon AK: **DNA-binding specificity of Mcm1: operator mutations that alter DNA-bending and transcriptional activities by a MADS box protein. Molecular and cellular biology.** *Mol Cell Biol* 1997, **17:**1881-1889.

28. Kumar R, Reynolds DM, Shevchenko A, Shevchenko A, Goldstone SD, Dalton S: **Forkhead transcription factors, Fkh1p and Fkh2p, collaborate with Mcm1p to control transcription required for M-phase.** *Curr Biol* 2000, **10:**896-906.

29. Boros J, Lim FL, Darieva Z, Pic-Taylor A, Harman R, Morgan BA, Sharrocks AD: **Molecular determinants of the cell-cycle regulated Mcm1p-Fkh2p transcription factor complex.** *Nucleic Acids Res* 2003, **31:**2279-2288.

30. Teixeira MC, Monteiro PT, Sa-Correia I: **Predicting gene and genomic regulation in *Saccharomyces cerevisiae*, using the YEASTRACT database: A step-by-step guided analysis.** *Methods Mol Biol* 2016, **1361:**391-404.
